# Evaluation of coronary stents: A review of types, materials, processing techniques, design, and problems

**DOI:** 10.1016/j.heliyon.2023.e13575

**Published:** 2023-02-09

**Authors:** Fatemeh Ahadi, Mohammad Azadi, Mojtaba Biglari, Mahdi Bodaghi, Ali Khaleghian

**Affiliations:** a Faculty of Mechanical Engineering, Semnan University, Semnan, Iran; b Department of Engineering, School of Science and Technology, Nottingham Trent University, Nottingham, United Kingdom; c Department of Biochemistry, Semnan University of Medical Sciences, Semnan, Iran

**Keywords:** Vascular stents, Coronary artery, Design, Materials, Processing technique, Mechanism of expansion

## Abstract

In the world, one of the leading causes of death is coronary artery disease (CAD). There are several ways to treat this disease, and stenting is currently the most appropriate way in many cases. Nowadays, the use of stents has rapidly increased, and they have been introduced in various models, with different geometries and materials. To select the most appropriate stent required, it is necessary to have an analysis of the mechanical behavior of various types of stents. The purpose of this article is to provide a complete overview of advanced research in the field of stents and to discuss and conclude important studies on different topics in the field of stents. In this review, we introduce the types of coronary stents, materials, stent processing technique, stent design, classification of stents based on the mechanism of expansion, and problems and complications of stents. In this article, by reviewing the biomechanical studies conducted in this field and collecting and classifying their results, a useful set of information has been presented to continue research in the direction of designing and manufacturing more efficient stents, although the clinical-engineering field still needs to continue research to optimize the design and construction. The optimum design of stents in the future is possible by simulation and using numerical methods and adequate knowledge of stent and artery biomechanics.

## Highlights

• Evaluation of coronary stents was reviewed for types, materials, processing techniques, design, and problems.
• A useful set of information is provided to continue the research to design and manufacture more efficient stents.
• For an optimal stent, various geometric properties such as the length, thickness, and scaffold design are important.
• The topology optimization of stents is essential with objectives of the structural and hemodynamics characteristics.

## Graphical abstract

**Figure:**
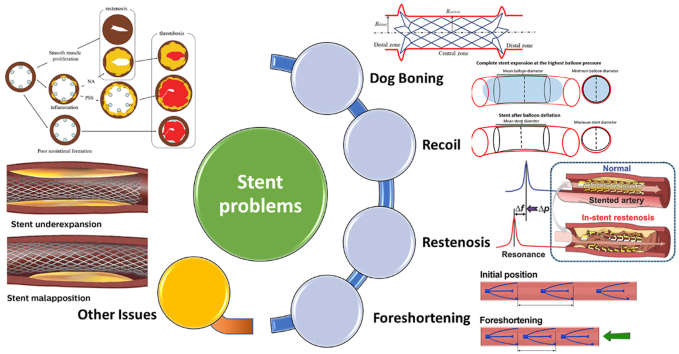

## 1 Introduction

Nowadays the world, coronary artery disease is one of the leading causes of human death, characterized by the narrowing of the arteries due to plaque [[1], [2], [3], [4], [5], [6]]. Atherosclerosis is a cardiovascular disease that has been on the rise worldwide in recent years. Its pathological mechanism is that fat or lipids accumulate on the artery wall under the influence of various cardiovascular risk factors. These deposits form a large number of plaques that lead to thickening of the arterial wall, blockage of blood vessels, and affect blood flow. Severe atherosclerosis can cause coronary artery disease, heart attack, and death [7,8]. There are several procedures for occluding arteries, including bypass, angioplasty by balloon, and stenting. Stenting is an important model in the treatment of atherosclerosis, which is performed to relieve obstruction and restore blood by placing a vascular stent on the stenosis and hard part of the artery, dilating at that point. Today, the use of cardiac stents has greatly increased due to their ease and efficiency in penetration. Therefore, the most appropriate stent design, stent evaluation, and analysis of different behaviors mechanically are very important [9]. Stents are small cylindrical scaffolds whose main purpose is to remove the arterial obstruction or to prevent elastic arterial reversibility following dilation of the artery or balloon angioplasty [10,11]. Stenting has advantages over other treatments such as no surgery, less complexity, less pain, and faster healing. Today, the use of coronary artery stents has increased. Coronary stents are currently used in more than 90% of PCI procedures [[12], [13], [14]].

Coated stents are new generation stents coated with a thin polymer membrane for vascular therapy. Coated stents increase tissue granulation and suppress thrombosis compared to bare-metal stents (BMS) [15].

Superficial features of coronary stents play an important role in inhibiting stent restenosis and also late stent thrombosis [16]. Coated stents are a new generation of stents that are coated with a thin polymer membrane to treat blood vessels. Coated stents increase tissue granulation and suppress thrombosis compared to bare-metal stents (BMS) [15,17]. In addition, optimized geometries for stents and surfaces, as shown by thin stents, help to decrease thrombosis, in spite of the stent configuration and variation in placement [17]. Stent thrombosis is a deadly complication. However, concerns that drug/polymer coatings are inherently thrombogenic should be reconsidered, as primary clotting by drug/polymer coatings is reduced [17].

Stent's efficiency metrics are longitudinal strength, radial strength, flexibility, fatigue resistance, tissue damage, drug distribution for drug-eluting stents, and flow criteria [18].

**Fig. 1:**
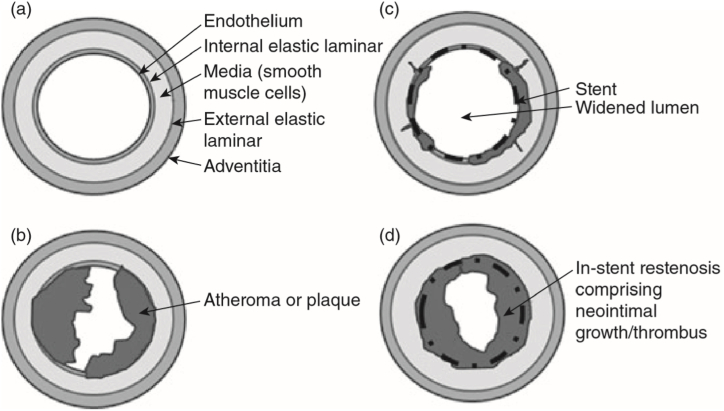
(a) Structure of an artery; (b) presence of atheroma; (c) enlarged lumen diameter after stenting; and (d) in-stent restenosis [25].

Stents can be categorized according to their expansion mechanism, their design, or by their materials [13,[19], [20], [21], [22]]. The results show that each stent topology has its own structural and hemodynamic function and the best stent can be selected according to the required clinical needs [23,24]. Fig. 1(a–d) show the structure of an artery before and after stenting [25].

In the present article, by reviewing the biomechanical studies performed in the field of optimal stent design, collecting information about different geometric properties of stents, and classifying their results, a set of information to continue research to design and the production of more efficient stents.

The review article includes important and discussed topics from the past to the present in the field of stents, and most of the purpose is to introduce these items next to each other and categorize them, directly from 117 sources given in the references.

The criterion and method of the summary of the review article are based on the classification of studies in different fields of stents, based on which it can be said that the breadth and variety in this field is one of the strengths of this field. In the following, these strengths and weaknesses in the field of stents are given.

## 2 History

**Fig. 2:**
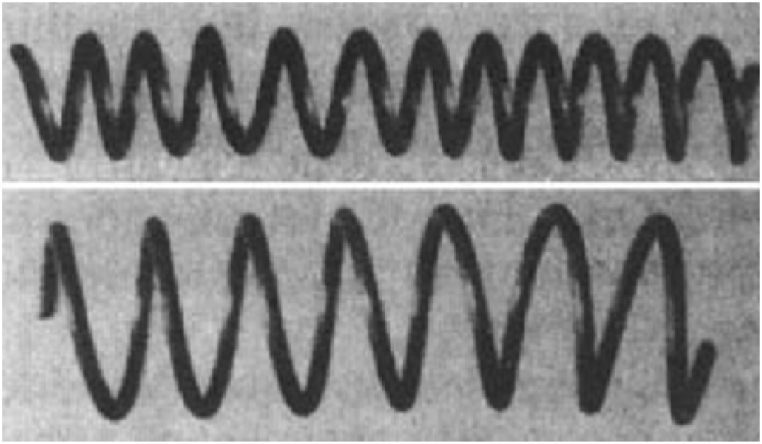
The stent designed by Dotter et al. [28].

Charles Theodore Dotter and Melvin P Judkins in 1964, presented the first angioplasty [26]. In 1969, Dotter, using stainless steel to wrap the coil stent, took the lead in researching the structure of vascular stents [[27], [28], [29]], Fig. 2 showed the stent designed by Dotter et al. [28].

Andreas Grantzyg in 1978, the first balloon angioplasty was done [30]. This led Puel and Sigwart [31] to discover the first coronary stent (in 1986), which was able to prevent acute vessel closure and late constrictive recoil [32]. Although many physicians subsequently made their stents, the first stent to be approved by the food and drug administration was made in 1987 [33]. Moreover, two important trials including the Belgian Netherlands stent test [34] and the stent restenosis study [35] were safe from stenting using dual antiplatelet therapy and appropriate implantation techniques [36,37]. In 2001, drug-eluting stents were introduced to minimize restenosis [38]. Since 2005, safety concerns about first-generation drug-eluting stents have increased [39,40], so new-generation stents have been developed [41]. Hence, new technologies for stents are constantly being discovered and supplied every day [42].

## 3 Strengths and weaknesses of stents

Studies in the field of stents from the past to today show that many useful topics have been addressed in the direction of its development. Therefore, by summarizing these studies, the strengths and weaknesses in this field and the design of stents are determined.

All these strengths and weaknesses in the studies can be seen in Table 1, and each of these topics has been examined in detail below.

**Table 1:** Strengths and weaknesses in studies on stents.

Strengths	Weaknesses
Variation in types of coronary stents	Restenosis
Variation in materials	Recoil
Variation in processing techniques	Dog boning
Variation in design	Foreshortening
Variation in the mechanism of expansion	

## 4 Strengths in studies on stents

As mentioned in Table 1, the general strength points in the field of stents include the variety in types of coronary stents, materials, processing techniques, designs, and mechanism of expansion. They all are summarized and put together in this review article.

Each of these topics is discussed in detail below.

### 4.1 Types of coronary stents

In general, there are three major types of coronary stents available today that are implanted in coronary arteries through angioplasty or percutaneous coronary intervention, consisting of [[43], [44], [45], [46], [47], [48], [49], [50]].

• Bare-Metal Stent (BMS)
• Drug-Eluting Stent (DES)
• Bioresorbable Vascular Scaffold (BVS)

#### 4.1.1 Bare-metal stents

Today, coronary stents have become the most usual treatment for coronary artery disease. Bare-metal stents have high intracoronary stent restenosis (ISR) rates [51]. In past studies, intracoronary stent restenosis was reported to be between 10% and 20% during a six-month follow-up, leading to myocardial infarction and angina, which required re-vascularization [52]. Healing with bare-metal stents have very beneficial results, and restenosis blood vessels were seen in 20–30% of patients in the span of 6–12 months [35]. Primary stents are made of stainless steel [53]. Stainless steel was replaced with chrome, cobalt, or other elements to retain strength [41,54].

#### 4.1.2 Drug-eluting stents

The advent of drug-eluting stents is considered a breakthrough. Biodegradation is considered a critical factor in establishing the efficiency of these stents [55].

The main purpose of a drug-eluting stent is to reduce intracoronary stent restenosis by inhibiting neointimal hyperplasia. Numerous agents affect drug elution from polymers, and how better drug release and the duration of drug elution are the most important issues in drug-eluting stent design [53]. A drug-eluting stent is associated with a reduced risk of restenosis [[56], [57], [58], [59]].

The superiority of drug-eluting stents over bare-metal stents with the recorded argument in ISR rates of somewhere in the span of 60%–80% [[60], [61], [62]]. Despite the achievement of DESs within the decrease of the ISR rate, issues have emerged over the long-time bio-compatibility of these devices because of cases of late destructive medicinal activities including stent thrombosis [63].

Traditional drug-eluting stents often delay endothelialization whilst inhibiting intimal hyperplasia [64]. In the literature [65], an arsenic trioxide-eluting stent effectively facilitates rapid re-endothelialization whilst preventing in-stent restenosis.

#### 4.1.3 Bioresorbable stents

In the body, there is increasing pressure on temporary implants, which requires the implanted material to be predictable over time to disappear. Such substances are called bioabsorbable [66]. The success of absorbable vascular scaffolding depends on the time of destruction [67]. Biodegradable stents are made of materials that can be broken down and absorbed by the body. The idea of the bioresorbable stent is considered revolutionary according to Erne et al. [68]. Numerous published studies center on the clinical, technological, and material features of bioabsorbable stents [33,53,[69], [70], [71], [72]]. Manufacturing of such these implants could be considered based on the applications for biocompatible biodegradable metals and alloys and also polymers which can be reabsorbed after several months of implantation, in the body [33,73,74]. The characteristics and degradation behavior of bioabsorbable stents must be predictable over some time. Ideally, bioabsorbable stents implanted in a vessel lack mechanical patronage for the recovery course and disappear within a few months (12–24 months) [75].

### 4.2 Materials

The materials used for coronary stents have evolved rapidly since the first stainless steel devices were introduced in 1987 [76].

Recent advances in the production of additives and the development of materials have led to four-dimensional (4D) printing technology to create dynamic devices that can change their shape and function on demand and over time. Recently, the possibility of 4D printing for some active materials with initial questions and challenges has been demonstrated [[77], [78], [79]].

The mechanical properties of some of the materials used to make stents are given in Table 2 [[80], [81], [82], [83], [84], [85], [86], [87], [88]]. Biodegradable stents do not suffer from fatigue due to their relatively short life and have the mechanical properties required for vascular spasms. They will also prevent future treatment bans. Pharmaceutical stents or drug-coated metal stents are some of the most popular types of stents today. These stents, which are more expensive than regular metal stents, prevent the re-growth of the vessel wall, resulting in the re-occlusion of the arteries [80].

**Table 2:** Mechanical properties of some materials utilized for fabricating stents.

Stent Material	Yong's Modulus (GPa)	Tensile Strength (MPa)	Elongation (%)	Degradation	References
Metals and alloys					
Nitinol	45–50	1200	∼20	–	[81,82]
Stainless steel	193	595	40	–	[81,83]
Cobalt-chromium MP35N	233	930	45	–	[81,84]
WE 43 Alloy (extruded)	44.2	280	2	1.35 mm/y	[85]
Biodegradable					
PLA	2–4	65	2–6	18–30 months	[86,87]
PLLA	2–4	60–70	2–6	>24 months	[86]
PGA	6–7	90–110	1–2	4–6 months	[86]
PCL	0.34–0.36	23	>4000	24–36 months	[86]
PAE	0.14–1.4	25–27	–	9–12 months	[85]
PTD-PC	1.2–1.6	60–220	–	6–48 months	[85]
TD-PCP	–	10–30	10–13	7 months	[88]

### 4.3 Stent processing technique

**Fig. 3:**
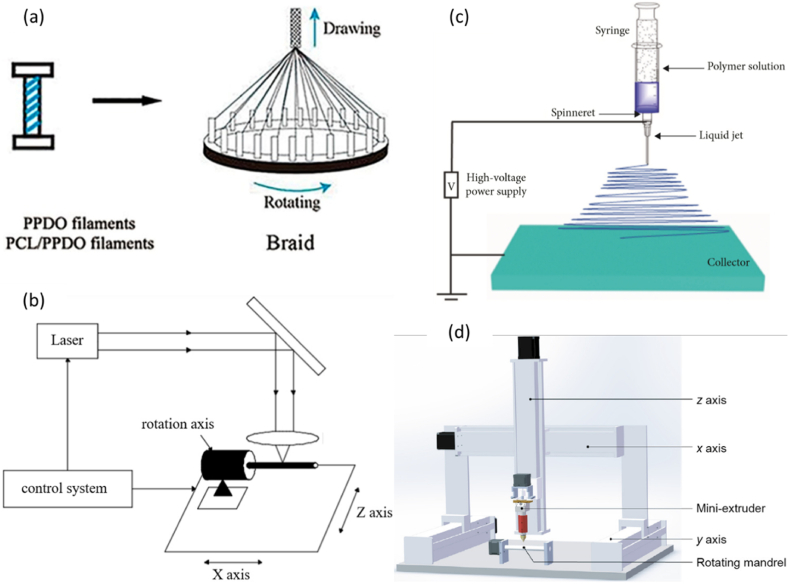
Vascular stent processing method: (a) braided [91]; (b) laser cutting [91]; (c) electrospinning technology [92]; (d) additive manufacturing [93].

The primary techniques for creating stents are the braided technique, laser slicing technique, electrospinning generation, and additive production generation (Fig. 3(a–d)) [[89], [90], [91], [92], [93]]. Fig. 3 suggests the techniques for processing vascular stents. In other words, Fig. 3(a) presents braided stents [91], Fig. 3(b) depicts laser cutting stents [91], Fig. 3(c) demonstrates the electrospinning technology [92], and Fig. 3(d) proposes additive manufacturing [93] for vascular stent processing techniques.

The braiding technique is that the wire is wrapped around the carrier and then the wire is woven along the axis of rotation in the prepared path to make a lattice stent. As the shape of stents turns into extra complicated today, the stent produced through the woven technique is constrained to an easy shape and the stent has a vulnerable radial stiffness. For this reason, the braiding technique is more suitable for making more compatible memory polymer stents [91].

Laser cutting is the most common method used to make a vascular stent. During the laser cutting process, a high-power laser canvas focuses on the tubular material, the material melts, evaporates, or wears out rapidly, and then the material is blown away by high-velocity airflow [91].

Electrospinning is a unique technique using an applied voltage for the liquid atomization process. The electrospinning technique undergoes fast development in recent years. This technique can provide the unlimited potential to achieve vascular graft prostheses. As shown in Fig. 3(a–d), first, a charged jet is created from a polymer solution using an electric field, after that, the solvent evaporates leaving behind a charged fiber that can be electrically deflected or collected on a metal substrate. Electrospinning generation isn't appropriate for getting ready complicated stent structures [92].

Additive production is very common today. Most researchers, in particular, use fused deposition modeling (FDM) to fabricate and reinforce stents. Screw extrusion-based 3D printing system consists of three main components: x-y-z motion system, rotation axis, and mini-screw extruder. In this technique, the printed filament is melted at excessive temperature and sprayed through the nozzle, and placed on a rotating shaft to hold the stent together. Because the additive fabrication generation capabilities a complicated printable shape for several sizes, it's been extensively utilized in-stent fabrication [90,93].

### 4.4 Stent design

The ideal stent should meet a broad span of technical versions. Stents should be secure and flexible enough to facilitate delivery to the lesion site. After expansion, the stents should apply enough radial force to the vessel wall to prevail the lesion resistance and elastic recoiling. Attempting to optimize one feature of one stent design may have damaging effects on another. Therefore, the best stent required should be used according to the required features [94]. Several parameters are significant in the design of stents, which are introduced in Table 3 [[95], [96], [97], [98], [99], [100], [101], [102], [103]] and explained in detail in the following.

**Table 3:** Important parameters in the design of stents.

Design parameters		References
Geometric parameters	Strut length	[95,102]
	Area	[95,102]
	Shape of strut cross-section	[95,102]
	Strut angle	[95]
	Stent links	[[96], [97], [98],^102^]
Flexibility		[96,100,101]
Radial stiffness		[96,100,101]
Concentration of tension		[103]
Fatigue		[103]

#### 4.4.1 Geometric parameters

**Fig. 4:**
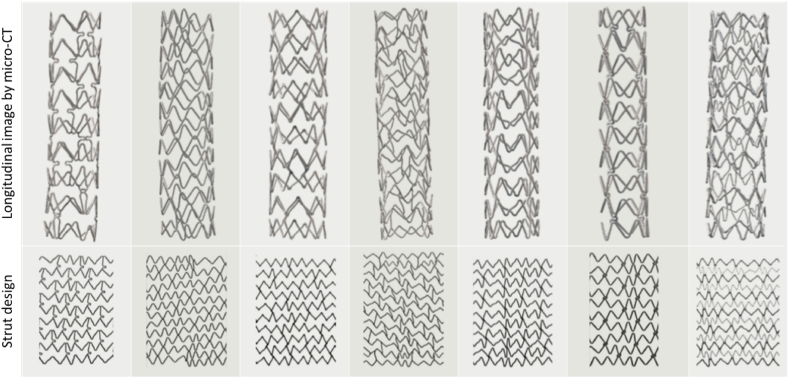
Micro-CT images of stents and the strut design [95].

Stent geometry design parameters can include strut length, area and shape of strut cross-section, and strut angle. In fact, by changing these parameters, stents will exhibit different mechanical behaviors. Fig. 4 shows some stents [95].

**Fig. 5:**
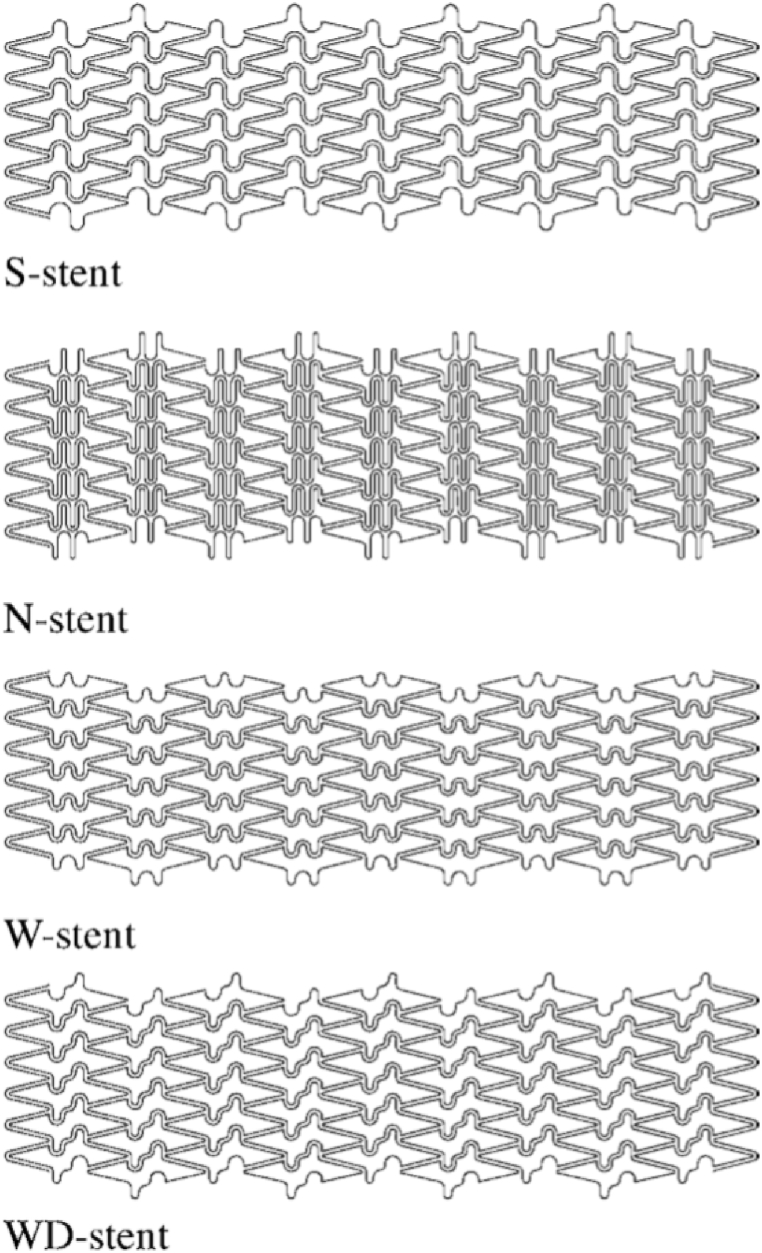
The sample of stent links [96].

The more symmetrical the design of the stent links, the more flexible it is. In Fig. 5, the stent is illustrated with different links (S-shaped, N-shaped, W-shaped, WD-shaped), where the W-shaped link has more flexibility due to symmetry, see Table 4 [[96], [97], [98]].

**Table 4:** Comparison of vascular stents with different links.

Stent links	Advantages	Disadvantages	References
S-shaped bridge	Axial flexibility	–	[97]
N-shaped bridge	–	Axial flexibility	[98]
W-shaped bridge	Bending stiffness	–	[96]
WD-shaped bridge	–	Axial flexibility	[96]

**Fig. 6:**
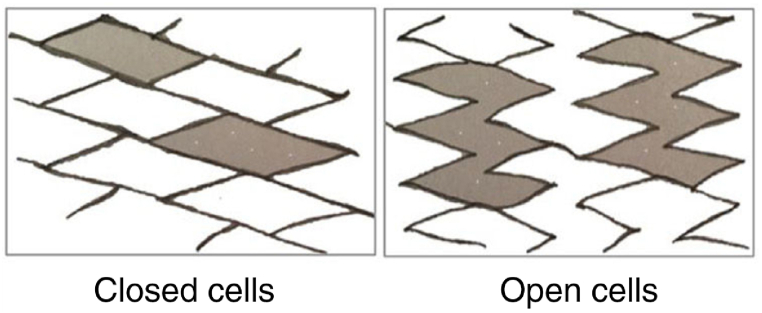
Open-cell and closed-cell shapes [102].

The stent can have several different modes in terms of the wire structure. The open-cell type consists of several welded loops and is more flexible than cell stents. Closed-cell stents are grooved tubes that are laser-shaped and have a higher radial stiffness than the open-cell model [99] (Fig. 6). There is also a combination of these two modes, which is called a type of hybrid cell that has both radial stiffness and good flexibility.

Closed-cell stents are less flexible and may have incomplete complexity and expansion. However, stents fit the open-cell formation best with angled vessels or twisted anatomy. The differences in the functional characteristics of the stent subgroups are specifically about the rate of free cell surface area among the scaffold ingredients [100].

The literature [101] states that carotid velocity increases disproportionately after coronary artery stenting (CAS) with closed-cell stents compared to open-cell stents. This issue suggests that the criteria of the velocity for quantifying stenosis may require a modification based on the stent design.

#### 4.4.2 Flexibility and radial stiffness

Radial flexibility and rigidity are important features in stent design [101]. For better performance, stents require high radial stiffness and at the same time a lot of flexibility, which is inversely related to each other. Flexibility is required to deliver the stent to the site of occlusion and high radial stiffness after the stent placement steps to prevent recurrent stenosis. The stent should be designed to show the desired feature at a specific time. For this purpose, the design is done in such a way that the stents have high radial stiffness by changing the angle during delivery, high flexibility, and the vertical and fixed angle after placement in the obstruction. Stents with symmetrical link configurations have high flexibility [96].

#### 4.4.3 Concentration of tension and fatigue

One of the goals of optimal stent design is to increase its fatigue life. Stress factors include stent-vessel and balloon-vessel confrontation, as well as excessive pressure increase when the stent is opened. Among the critical areas for stress are areas close to the fork, the contact point of the stent slits, the end of the plate, the curved parts of the stent, as well as the corners, and the middle of the slit. Ways to reduce stress in the design include reducing the width of the stent wire, reducing the length of the gap, and the use of stents with a body or biodegradable coating [103].

### 4.5 Classification of stents based on the mechanism of expansion

**Fig. 7:**
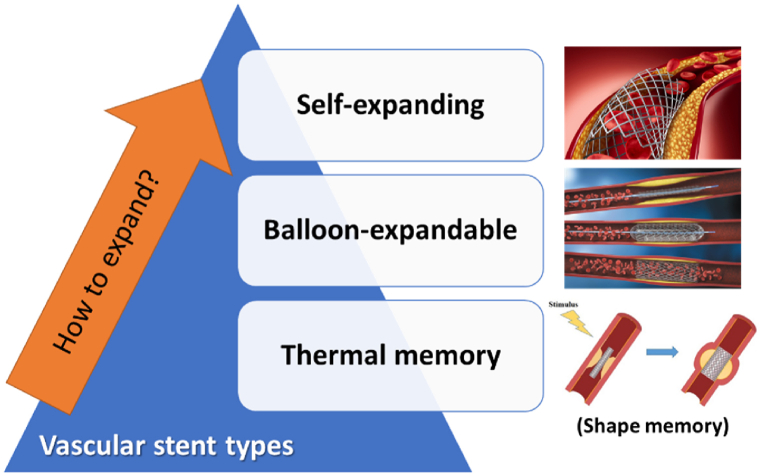
A classification of stents based on the mechanism of expansion.

Stents are classified into three categories based on the expansion mechanism, including balloon-expandable stents, self-expanding stents, and thermal memory stents [11,73,94,99,[104], [105], [106], [107], [108], [109]] (Fig. 7). Balloon-expandable and self-expanding and thermal memory stents have various mechanical properties and dynamics. Balloon-expandable stents attain their maximum diameter at the moment of implantation, self-expanding stents continue to expand after several days and reach their maximum diameter, just a few weeks after implantation [110].

#### 4.5.1 Balloon-expandable stents

**Fig. 8:**
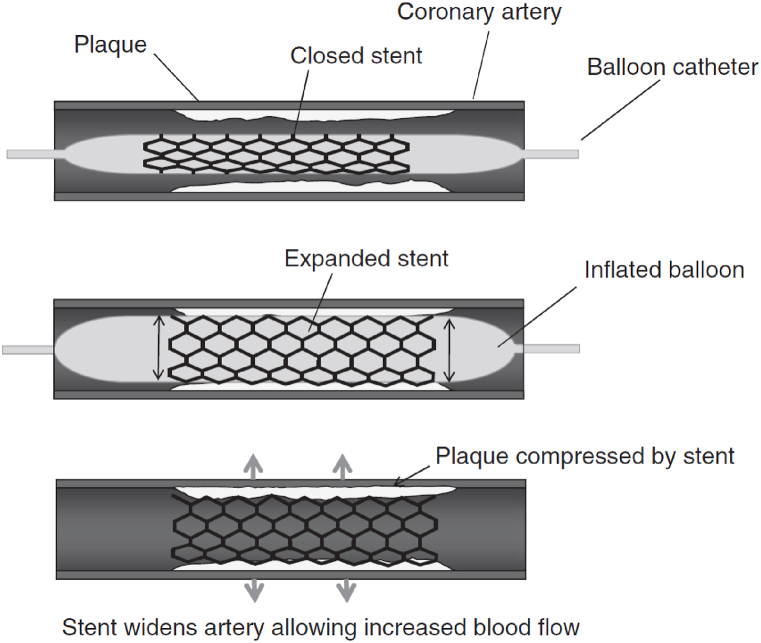
Balloon-expandable stents [114].

Balloon-expandable stents are mounted on a balloon in contraction mode. In balloon-expandable stents, the stent is placed with the delivery system in the desired location and then deployed by the expansion of an expanded balloon (Fig. 8) [104,111]. After the balloon expands, the stent must have sufficient radial strength as well as sufficient flexibility to accommodate the vessel [94,104,112,113].

#### 4.5.2 Self-expanding coronary stents

**Fig. 9:**
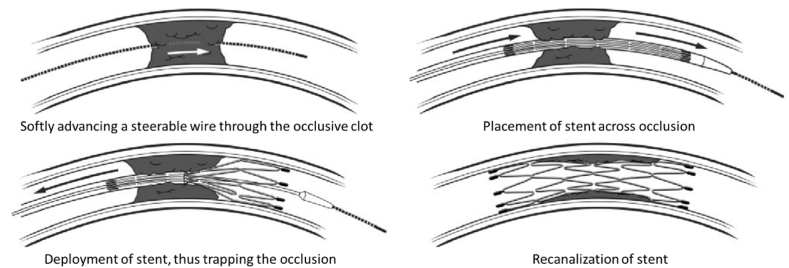
Steps involved in self-expanding stent delivery and deployment [115].

Self-expanding stents are springy and limited in diameter and reach their predetermined diameter by removing the restraints. Self-expanding stents are spring stents and are limited in diameter by the constraints and after being in the desired location, they reach their determined diameter by removing the constraints [104,111]. Fig. 9 shows the steps related to self-expanding stent delivery and deployment. The self-expanding stents have less axial stiffness and are therefore more flexible and more in line with the shape of the vessels than the shape of the stent [110]. Therefore, in some cases, balloon-expandable stents are not suitable and require self-expanding stents [104,114].

#### 4.5.3 Thermal memory stents

Thermal memory stents lose their shape with the application of heat and regain their shape in the biological environment, and in these stents, a nitinol (nickel, titanium) alloy that has a thermal memory is often used [108].

## 5 Weaknesses and problems in stents

Common complications of stents include tissue growth within the stent, recurrent vascular occlusion, thrombosis, stent failure, and vascular damage. Ways to reduce these problems include increasing radial stiffness, increasing fatigue life, reducing stent strain, and proper delivery mechanism, which are described in the following parameters.

**Fig. 10:**
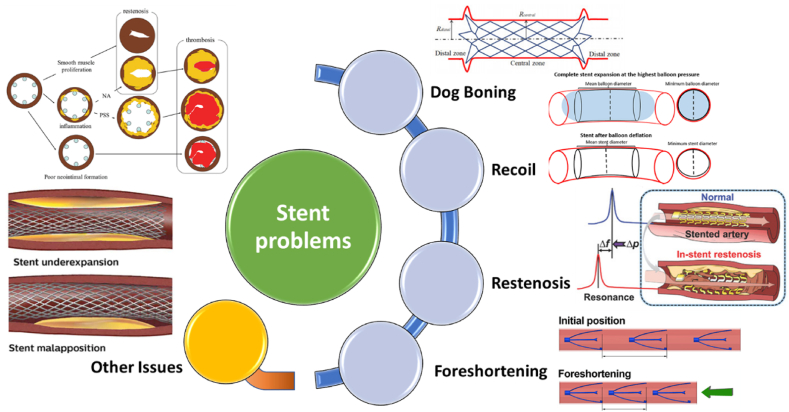
Problems of stents.

In studies in the field of stents, for the design of stents, weak points including restenosis, recoil, dog boning, and foreshortening have been pointed out (Fig. 10). Each of these topics is discussed in detail below.

### 5.1 Restenosis

The reduction of vessel diameter (more than 50%) after stenting is called recurrent stenosis, which will disrupt blood flow. This complication is more common in men than women and its causes can be low radial stiffness, stress concentration, and uneven distribution of struts in the stent, which will lead to increased hyperplasia and seizures. The likelihood of re-stenosis decreases. Critical areas for re-stenosis include bifurcations and branches and two ends of the stent [116].

### 5.2 Stent recoil

The stent usually shrinks slightly in diameter after the balloon expands and exits and recedes from its position. This phenomenon is described by *DRR* and *CRR* in Eqs. (1), (2) [94]:

(1) $$DRR=Distal\_Radial\_Recoil=\frac{R_{distal}^{load}-R_{distal}^{unload}}{R_{distal}^{load}}$$

(2) $$CRR=Central\_Radial\_Recoil=\frac{R_{central}^{load}-R_{central}^{unload}}{R_{central}^{load}}$$

where $R_{distal}^{load}$ radius after applying pressure at the end of the stent and $R_{distal}^{unload}$ radius after removal of the pressure at the end of the stent and $R_{central}^{load}$ radius after applying pressure to the center of the stent and $R_{central}^{unload}$ the radius after removal of the pressure from the part the center is the stent.

A smaller reduction of the diameter is better. Increased stent opening pressure is one of the factors that reduce the retraction and the ratio of a metal surface to low vessels, as well as the greater thickness of the stent, are the factors that increase this complication.

### 5.3 Dog boning

The further opening of the two ends of the stent relative to the central part and the creation of a radius difference of dog boning (*DB*) is called, which is shown by Eq. (3) [94]:

(3) $$DB=Dog\_Boning=\frac{R_{distal}^{load}-R_{central}^{load}}{R_{distal}^{load}}$$

where $R_{distal}^{load}$ is the radius of the end of the stent after pressure and $R_{central}^{load}$ is the radius of the center of the stent after pressure. This complication causes damage to the arteries and impaired blood flow, and the ways to reduce this phenomenon are to reduce the balloon length, increase the width of the ester and reduce the ratio of the metal surface to the arteries.

### 5.4 Foreshortening

As the diameter of the stent increases, its length decreases. This phenomenon is called Foreshortening, which is measured by Eq. (4) [94,117]:

(4) $$Foreshortening=\frac{L_{original}-L_{load}}{L_{original}}$$

where $L_{original}$ is the initial length of the stent and $L_{load}$ is the deployed length. This phenomenon can be reduced with bioabsorbable coatings. Naturally, the smaller the length of this reduction, the more desirable it is.

## 6 Future of stents

Angioplasty with a stent is a procedure to open narrowed or blocked blood vessels. Stenting is one of the most important strategies to remedy atherosclerosis. Moreover, the implantation of stents is a non-surgical technique, a less invasive procedure than surgery, to treat coronary artery disease that could reduce the risk of a heart attack. Implantation of the most appropriate stent is important to the patient to reduce the risk of complications. Researchers are working on ways to improve current stent angioplasty procedures or add adjunctive therapies. Innovations in angioplasty and stents continue for challenging treatments.

**Fig. 11:**
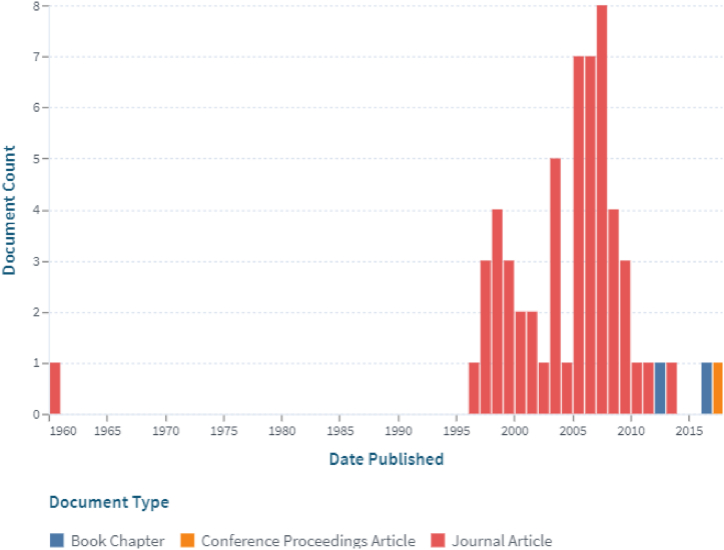
The working trend of documents on the stent (Lens Scholarly Search: Stent).

**Fig. 12:**
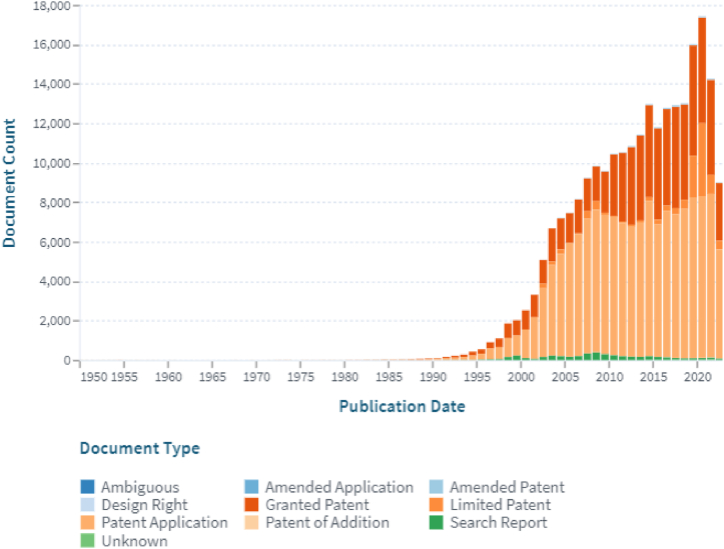
The working trend of patents on the stent (Lens Patent Search: Stent).

Finally, the word “stent” was searched on the web-based on lens site (www.lens.org) to obtain the document workflow, as shown in Fig. 11. There is also such a report for patents, which is shown in Fig. 12.

## 7 Concluding remarks

There are several ways to treat coronary artery disease, and stenting is currently the most appropriate way in many cases. Nowadays, the use of stents has increased rapidly, and stents have been introduced in various models, with different geometries and materials. The researchers successfully solved the difficulty of vascular occlusion with bare-metal stents and later developed a drug-eluting stent to minimize restenosis. They have also improved their coronary stents by improving the technology of environmentally absorbable scaffolding and possibly further development.

The advent of biodegradable stents that prescribe drugs represents a significant breakthrough in coronary artery disease, which represents a step towards a change in the pattern of the treatment.

Several stent features are related to the material selection, such as material types, material composition, and architectural features. In addition, each stent structure shows a different function, and according to the required clinical needs, the best stent can be selected.

For the future perspective, despite the progress of researchers in the proper design of stent geometry, the clinical-engineering field still needs to continue research to optimize the design and construction. Optimal design of stents in the future is possible by simulating and using numerical methods and sufficient knowledge of the biomechanics of stents and arteries. This goal could be obtained by the topology optimization of stents considering the fluid-solid interaction in arteries, with the objective of the structural and hemodynamics characteristics.

## Author contribution statement

All authors listed have significantly contributed to the development and the writing of this article.

## Funding statement

This research was supported by 10.13039/501100003968Iran National Science Foundation (10.13039/501100003968INSF) under project No. 4004237.

## Data availability statement

Data included in article/supp. material/referenced in article.

## Declaration of interest's statement

The authors declare that they have no known competing financial interests or personal relationships that could have appeared to influence the work reported in this paper.

## Abbreviations

BMS: Bare-Metal Stent
BVS: Bioresorbable Vascular Scaffold
CAD: Coronary Arteries Disease
CAS: Coronary Artery Stenting
CRR: Central Radial Recoil
DB: Dog Boning
DES: Drug-Eluting Stent
DRR: Distal Radial Recoil
FDM: Fused Deposition Modeling
ISR: Intracoronary Stent Restenosis
PCI: Percutaneous Coronary Intervention
